# Research on the impact of the digital economy on carbon emissions based on the dual perspectives of carbon emission reduction and carbon efficiency

**DOI:** 10.1038/s41598-025-87098-1

**Published:** 2025-01-27

**Authors:** Xin Liu, Liang Chen, Yifu Lu, Ming Chang, Yi Xiao, Haonan Yang, Deyuan Kong, Lili Zhang

**Affiliations:** 1 School of Finance and Accounting, Chengdu Jincheng College, Chengdu, 610097 Sichuan China; 2 College of Business and Economics, Australian Capital Territory , Australian National University, Canberra, 2600 Australia; 3 State Key Laboratory of GeoHazard Prevention and GeoEnvironment Protection, Chengdu University of Technology, Chengdu, 610059 Sichuan China; 4 College of Business, Chengdu University of Technology, Chengdu, 610097 Sichuan China; 5 College of Business, Southwest Jiaotong University Hope College, Chengdu, 610400 Sichuan China

**Keywords:** Digital Economy, Carbon emissions, Carbon Emission reduction, Carbon Efficiency Enhancement, Environmental economics, Sustainability

## Abstract

China’s digital economy is currently thriving, with the “dual carbon” targets representing a significant pursuit of economic development. The role of the digital economy in achieving these targets warrants detailed discussion. Using urban panel data from China spanning 2011 to 2021, this paper empirically examines the impact of the digital economy on urban carbon emissions. The findings reveal several key points: Firstly, the digital economy significantly reduces urban carbon emissions and enhances efficiency, a conclusion that remains valid after a series of robustness checks. Secondly, there is a notable structural effect, with different dimensions of the digital economy exhibiting varying impacts on urban emission reduction and efficiency enhancement. Thirdly, green economic efficiency and green technological innovation are crucial mechanisms through which the digital economy facilitates urban carbon emission reduction and efficiency improvement, operating through green development and innovation channels. Fourthly, industrial structure upgrading is a key nonlinear factor reducing the digital economy’s impact on carbon reduction, while the digital economy’s effect on urban carbon efficiency shows a U-shaped pattern. Lastly, the impact of the digital economy on urban carbon emissions displays significant heterogeneity across cities of different locations, tiers, and characteristics.

## Introduction

Currently, global warming poses economic, social, and environmental challenges worldwide, primarily due to greenhouse gases, especially carbon dioxide. The World Meteorological Organization’s (WMO) “Global Climate Report 2023” states that 2023’s global average temperature was 1.4 °C above pre-industrial levels (1850–1900), marking it the warmest year in 174 years. Meanwhile, the International Energy Agency’s (IEA) “CO2 Emissions in 2023” report shows a 1.1% increase in global CO2 emissions. To tackle warming, achieve sustainable development, and demonstrate responsibility, China has proposed “dual carbon” targets: “carbon peaking” and “carbon neutrality,” aiming for a low-carbon, green economy, ensuring growth quality, and contributing to global carbon reduction.

In this context, China’s “dual carbon” commitment to humanity and carbon emission reduction strategies have become key research topics. Concurrently, the IT revolution has propelled the digital economy’s vigorous growth. According to the “China Digital Economy Development Research Report (2023)” by the China Academy of Information and Communications Technology (CAICT), China’s digital economy hit 50.2 trillion yuan in 2022, surpassing nominal GDP growth for 11 years. It now comprises 41.5% of GDP, matching the secondary industry’s share, highlighting its key role in national economic development. Of particular note is the close and intricate relationship between the digital economy and urban carbon emissions. On one hand, the digital economy contributes to reducing urban carbon emissions by enhancing energy efficiency, promoting advancements in low-carbon technologies, and fostering green innovations^1^. On the other hand, it may indirectly lead to increased carbon emissions due to energy consumption in related industries such as data center operations and e-commerce logistics^2^. Consequently, the impact of the digital economy on urban carbon emissions is multidimensional, nonlinear, and shaped by numerous factors.

Given that cities are the primary sources of global carbon emissions, it is particularly crucial to deeply explore the complex interplay between the digital economy and urban carbon emissions. Under the framework of the “dual carbon” targets, investigating the impact of the digital economy on urban carbon emissions not only aids in enhancing our understanding of this domain but also provides new insights and methodologies for carbon reduction and efficiency improvement, thereby promoting harmonious development among the digital economy, carbon reduction, and urban efficiency. This research holds significant importance for guiding holistic urban management, optimizing carbon emission control strategies, formulating macro-policies, and addressing scientific issues.

## Literature review

### Existing research on the digital economy and carbon emissions

With the introduction of “dual carbon” goals and the thriving digital economy (DE), scholars have increasingly focused on its energy-saving and emission-reduction effects. Huang^3^ asserts that achieving these goals necessitates a comprehensive systemic transformation involving various aspects, with carbon-neutrality science and technology serving as the cornerstone. In the context of global climate change, carbon emissions (CE) have garnered significant attention, and DE has emerged as a novel perspective for carbon reduction research. Recent studies have examined the relationship between DE and CE from various angles, yielding mixed results^4^. Xie et al. developed an index system for digital economy (DE), revealing a relationship with urban carbon emissions (CE) that initially grows but later substantially reduces. Some studies suggest that DE development facilitates CE reduction, especially in China and developing countries^5–7^. , with the emission reduction effect becoming more pronounced as DE matures. Spatial spillover effects on CE reduction have also been observed, characterized by regional heterogeneity, such as more significant reduction in eastern China^36^. However, contrasting viewpoints argue that DE exacerbates CE due to significant emissions from the Information and Communication Technologies (ICT) sector, which requires high carbon-emitting intermediate inputs, indirectly increasing energy demand and hindering carbon reduction efforts^2^.

### Multi-dimensional indexes and their impact on carbon emissions

Several studies have constructed multi-dimensional indexes to assess the digital economy’s impact on carbon emissions. Chang et al.^8^ created a four-dimensional index, revealing a significant reduction in regional carbon emission intensity, with industrial structure upgrading as a key mechanism. Li et al.^9^ measured digital economy development through internet and digital finance, demonstrating its positive impact on regional carbon reduction, highlighting green technological progress as a crucial factor. Wang^10^ established an index system encompassing digital industrialization and industrial digitization, finding that urban carbon emissions first increase and then decrease, with green technological innovation and industrial structure upgrading as significant pathways. Zhang et al.^11^ created a digital economy index covering digital industry, innovation, and application, showing a positive impact on urban carbon emission performance. Li et al.^12^ assessed urban digital finance development, finding it significantly enhances urban industrial carbon emission efficiency, with green technological innovation as a critical threshold factor. Detailed analysis is provided on the structural effects of various digital economy dimensions, aligning with the emphasis on smart city technology for emission management by He et al.^13^.

### Literature deficiencies and paper contributions

Existing literature has provided valuable insights into the relationship between the digital economy and carbon emissions, yet several deficiencies persist. Typically, studies focus solely on emission reduction or efficiency enhancement, neglecting a comprehensive analysis that integrates both aspects. Furthermore, the influence of technical attributes on carbon emissions is often overemphasized, while the perspective of green economic development is overlooked. The measurement of the digital economy is limited to two to three dimensions, lacking a holistic assessment of its various impacts. Additionally, most research explores linear relationships, disregarding potential nonlinear interactions.

This paper endeavors to address these deficiencies and further explores the following aspects: Firstly, it focuses on the dual-carbon goals, examining the impact of the digital economy on urban carbon emissions from both carbon reduction and efficiency perspectives. Secondly, it broadens the scope beyond green technology, exploring the mechanisms of the digital economy through green development and innovation, with empirical tests demonstrating the role of green technological innovations in enhancing carbon reduction and efficiency. Thirdly, based on the definitions and research of the China Academy of Information and Communications Technology (CAICT), a new index system is constructed to measure digital economy development across four dimensions, incorporating both economic and social welfare into carbon emission assessments. Fourthly, by considering industrial structure upgrading as a threshold variable, this paper deepens the understanding of the nonlinear relationship between the digital economy and carbon emissions from both reduction and efficiency angles. Lastly, a geographical and time-weighted regression (GTWR) analysis is conducted, with the carbon emission coupling coordination degree (syeff) as the dependent variable and the digital economy (Dige) as the explanatory variable.

## Theoretical basis and research hypotheses

### Direct impact of the digital economy on urban carbon emissions

On one hand, the digital economy reduces urban carbon emissions by integrating with the urban economy, promoting industries like AI, machine learning, and the metaverse, and shifting urban industries to high-tech, low-carbon sectors (Shi)^14^. Additionally, the development of the digital economy has spawned new models like the sharing economy and platform economy (Pouri and Hilty)^15^, optimizing resource utilization efficiency, eliminating redundant capacity, and further reducing overall urban carbon emissions. Secondly, the widespread adoption of the digital economy at the enterprise level has introduced digital production and operation methods, including smart manufacturing, remote office operations, and supply chain optimization in logistics and transportation. These advancements have spurred innovative developments in production methods and business models, curbing energy consumption and carbon emissions during traditional enterprise operations (Han et al.)^16^. Thirdly, the extensive application of the digital economy in residents’ lives has promoted the popularization of new intelligent and digital lifestyles, including e-commerce, smart homes, and intelligent transportation (Ban and Zhang)^17^. This has effectively decreased energy consumption in traditional retail and contributed to lowering carbon emissions in residents’ daily lives. Lastly, the extensive utilization of the digital economy in government administration enables government departments to monitor and manage urban energy consumption and carbon emissions through digital city management systems (Zhang et al.)^18^. This facilitates the implementation of policies and initiatives such as low-carbon transportation, energy-efficient buildings, and green production, further reducing urban carbon emissions.

On the other hand, the direct impact of the digital economy on urban carbon emissions is also manifested in terms of efficiency enhancement. Shen, Y. et al.^19^ insights underscore the potential of policy strategies that leverage the digital economy and technological innovation to meet the “dual-control” policy objectives and foster sustainable development. Firstly, the rapid development of the digital economy has fostered the emergence and growth of high-tech, green, low-carbon, and energy-saving technologies (Shao et al.)^20^. The application of these technologies across various sectors of the economy has improved resource utilization efficiency, driven economic growth through innovation, and increased economic output per unit of carbon emission, effectively enhancing the economic performance of carbon emissions. Secondly, while reducing urban carbon emissions, the digital economy has also promoted innovative development in the healthcare sector, facilitated the popularization and optimization of educational resources, and created more job opportunities and positions. These advancements have effectively improved urban healthcare, education, and income levels, enhancing the welfare performance of urban carbon emissions (Wang et al.)^21^. Based on the above, the following hypotheses are proposed:

#### H1

The digital economy has a mitigation effect on urban carbon emissions.

#### H2

The digital economy has an efficiency enhancement effect on urban carbon emissions.

### Indirect impact of the digital economy on urban carbon emissions

1. Green Development Effect.

The digital economy, driven by data and technology, has become crucial for green development. Firstly, the digital economy advances intelligent manufacturing like industrial internet, big data analytics, and AI, enhancing production efficiency and greenness(Lyu et al.)^22^. Secondly, digital tech in energy systems boosts smart energy management, optimizing energy use and enhancing efficiency via real-time monitoring (Wang et al.)^23^. Lastly, digital economy drives online consumption patterns, promoting low-input, low-energy, low-pollution services like logistics, enhancing green consumption (Ban and Zhang)^17^. Consequently, the development of the digital economy contributes to the improvement of urban green economic efficiency from both the production and consumption ends, fostering the development of a green economy.

Furthermore, the enhancement of urban green economic efficiency has a direct and profound impact on urban carbon emission reduction and efficiency enhancement. Firstly, enhanced green economic efficiency leads to more efficient resource use in production and consumption (Li et al.^24^, reducing waste and carbon emissions. For example, energy-efficient tech cuts firm energy use and emissions, while digital platforms help residents reduce offline consumption emissions. Secondly, enhanced green economic efficiency maximizes resource utilization (Lin and Tan)^25^, leading to greater economic output per unit of carbon emission, effectively improving the economic performance of carbon emissions. Lastly, the improvement in green economic efficiency fosters the development of numerous green technologies and services, creating more job opportunities and related positions, generating social employment and income effects. Additionally, the enhancement of green economic efficiency enables cities to achieve green economic development (Lou et al.)^26^, signifying improved environmental quality and reduced pollution, leading to positive social health effects. Therefore, the improvement in green economic efficiency also has a positive impact on the social welfare performance of carbon emissions. Based on the above analysis, the following hypothesis is proposed:

#### H3

The digital economy can form a green development effect by enhancing urban green economic efficiency, thereby exerting a carbon emission efficiency enhancement effect on cities.

(2) Green innovation effect.

Minimizing carbon emissions and enhancing carbon efficiency both require a technological foundation, particularly green technological innovation supported by green technologies. This innovation necessitates funding, resources, talent for R&D, and a large market for promotion and sales. The digital economy offers safeguards for this (Dian et al.)^27^. Specifically, firstly, the digital economy offers abundant data resources and information technology support for green technological innovation (Chen et al.)^28^. Through big data analysis and artificial intelligence, environmental issues and carbon emission sources can be more accurately identified, providing data support and scientific basis for the R&D and application of green technologies. Furthermore, the digital economy facilitates the integration and innovation of green technologies, promoting cross-sectoral applications and development. Secondly, the digital economy introduces new investment and financing models and platforms, such as digital inclusive finance and Ant Financial Services, providing more flexible and diversified funding support for the R&D and application of green technologies (Xu et al.)^29^, lowering the financing threshold for green technological innovation. Thirdly, the digital economy actively integrates with the education sector, cultivating a large number of digital talents for economic and social development, providing corresponding talent support for green technological innovation (Huang et al.)^30^. Lastly, the development of the digital economy drives the growth of emerging industries and future industries, expanding the market space and application scope of green technologies (Lou et al.)^31^. Concurrently, it promotes the integration and application of green technologies with traditional industries, advancing the marketization and industrialization of green technologies.

Furthermore, the enhancement of urban green technological innovation has a direct and profound impact on urban carbon emission reduction and efficiency enhancement. Firstly, green technological innovation contributes to the low-carbon transformation of production and consumption processes. It enables the low-carbon upgrading of production processes, machinery and equipment, consumption platforms, and consumption scenarios, promoting technological innovation, management optimization, and equipment upgrades (Xu et al.)^32^, forming green supply chains, thereby reducing carbon emissions during production and consumption and achieving bi-directional carbon emission reduction at both the source and the end. Secondly, green technological innovation can reduce production costs for enterprises. Traditional production methods often rely on high energy consumption and inputs, whereas the introduction of green technologies enables enterprises to reduce energy consumption and resource inputs (Du et al.)^33^, thereby lowering production costs and improving the economic performance of carbon emissions. Moreover, after adopting green technologies, enterprises can enhance their green production capabilities, produce environmentally friendly products that meet market demands, improve market competitiveness, increase market share, and further elevate the economic performance of carbon emissions. Lastly, the application of green technological innovation in various aspects of social production and life contributes to improving urban living quality, enhancing urban environments, and raising urban landscape quality, thereby elevating residents’ happiness and satisfaction with their living and consumption, and improving the social welfare performance of urban carbon emissions (Tang et al.)^34^. Based on the above analysis, this paper proposes the following hypothesis:

#### H4

The digital economy can form a green innovation effect by enhancing urban green technological innovation, thereby exerting a carbon emission efficiency enhancement effect on cities.

### Nonlinear effects of the digital economy on urban carbon emissions

Numerous studies have highlighted the pivotal role of industrial structure upgrading in mediating the impact of the digital economy on carbon emissions (Yi et al.; Dong et al.)^35,36^. However, most studies have focused on the linear impact of industrial structure upgrading on urban carbon emissions influenced by the digital economy, following the pathway of digital economy → industrial structure upgrading → carbon emissions. Nevertheless, while industrial structure upgrading often entails the expansion of high-tech, emerging, and future industries, it can also encompass carbon-intensive sectors such as chip production and electronics manufacturing within the electronic information industry, which may contribute to increased carbon emissions. Furthermore, during the process of industrial restructuring, while traditional industries gradually decrease their output and new industries increase theirs, the continued operation of equipment and factories from traditional industries can lead to an inertial effect on carbon emissions during the transition period (Wang et al.)^37^, thereby elevating overall carbon emissions. Additionally, the initial application of new technologies resulting from industrial structure upgrading may not be fully optimized, resulting in low energy efficiency and subsequently higher carbon emissions (Li et al.)^38^. Based on the above analysis, Hypothesis 5 is proposed:

#### H5

The impact of the digital economy on urban carbon emissions exhibits a nonlinear threshold effect contingent upon the level of industrial structure upgrading.

## Current situation analysis

This study employs the Arc-GIS tool and applies the natural breaks classification method to create distribution maps of China’s digital finance development level, carbon emission intensity, and carbon emission performance at the city level for the years 2011, 2016, and 2021, respectively.

**Fig. 1:**
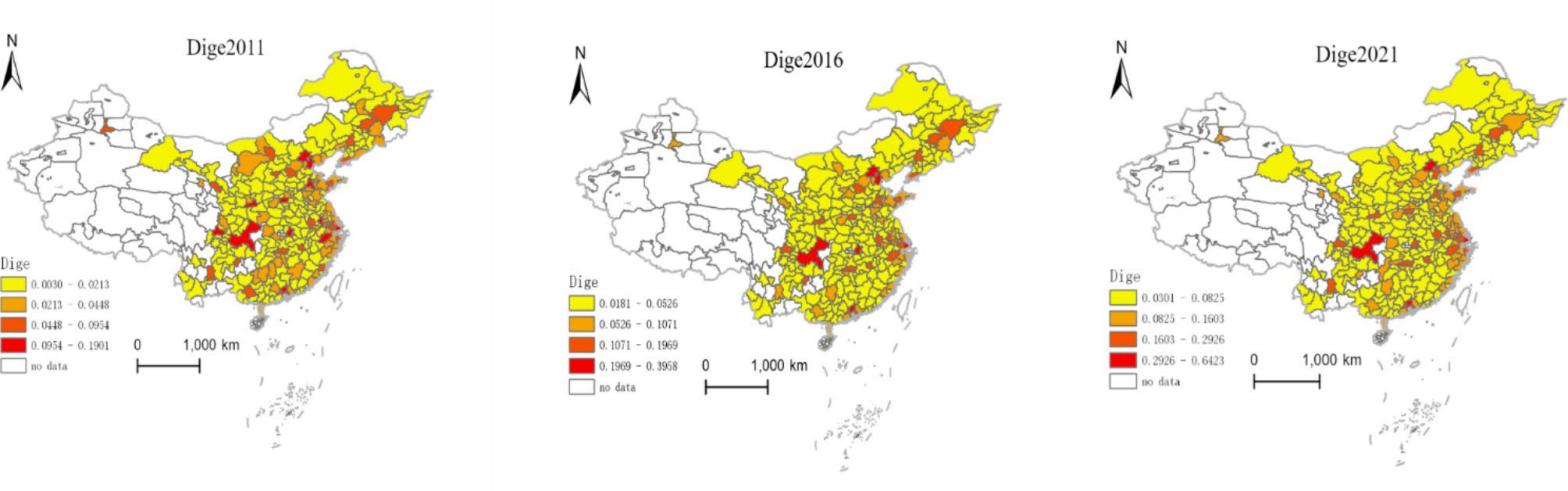
Distribution Map of Urban Digital Economy Development. Version: ArcMap 10.8 for desktop URL link: https://www.esri.com/en-us/home.

From Fig. 1, it can be observed that in 2011, the development of the digital economy generally showed higher levels in the east and south compared to the west and north. The regions with high levels of digital economy development are concentrated mainly in eastern coastal cities, while the central and western regions have relatively lower levels of digital finance development. In 2021, the overall pattern of higher levels in the east and south and lower levels in the west and north persisted, but compared to 2011, the digital economy development level in the central and western regions has seen marked improvements. Furthermore, examining the extreme values of digital economy development levels, in 2011, Guangzhou City in Guangdong Province had the highest level (0.19), while Longnan City in Gansu Province had the lowest (0.003), with a difference of approximately 63 times between the two cities. In 2021, Shanghai became the city with the highest level of digital economy development (0.64), while Guyuan City in Ningxia Hui Autonomous Region had the lowest (0.03), showing a difference of approximately 21 times. This indicates that over the years, the digital economy development level in China’s cities has steadily improved, and the spatial disparity in digital economy development among cities is gradually narrowing.

**Fig. 2:**
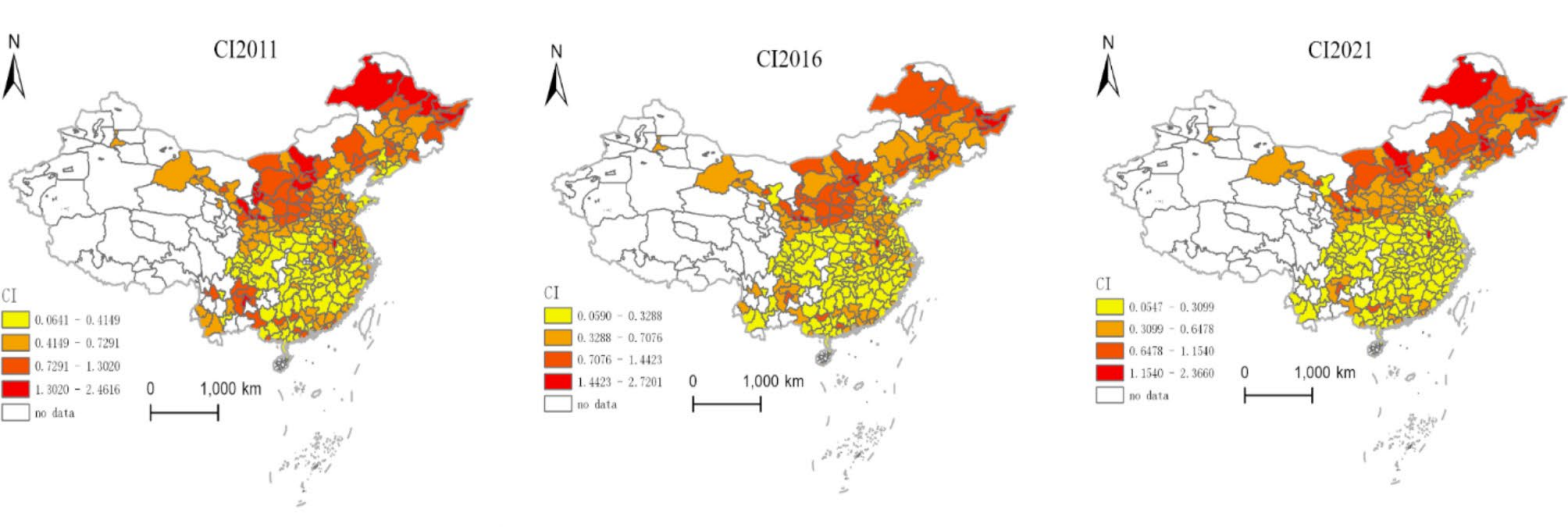
Distribution map of urban carbon emission intensity. Version: ArcMap 10.8 for desktop URL link: https://www.esri.com/en-us/home.

From Fig. 2, it is observable in 2011 that the overall carbon emission intensity generally shows higher levels in the north and south compared to the central region. The regions with high carbon emission intensity are concentrated mainly in the northern areas, while the central and southern regions have relatively lower carbon emission intensities. In 2021, the overall pattern of higher levels in the north and south and lower levels in the central region persisted, but compared to 2011, the carbon emission intensity in the northern regions decreased, with the most significant reductions seen in the Yangtze River Delta region and Yunnan-Guizhou areas. Additionally, examining the extreme values of carbon emission intensity, in 2011, Datong City in Shanxi Province had the highest carbon emission intensity (2.46), while Ziyang City in Sichuan Province had the lowest (0.06), with a difference of approximately 41 times between the two cities. In 2021, Qitaihe City in Heilongjiang Province became the city with the highest carbon emission intensity (2.36), while Bazhong City in Sichuan Province had the lowest (0.05), showing a difference of approximately 47 times. This indicates that over the years, the carbon emission intensity in China’s cities has decreased significantly, but the spatial disparity in carbon emission intensity among cities has gradually increased.

**Fig. 3:**
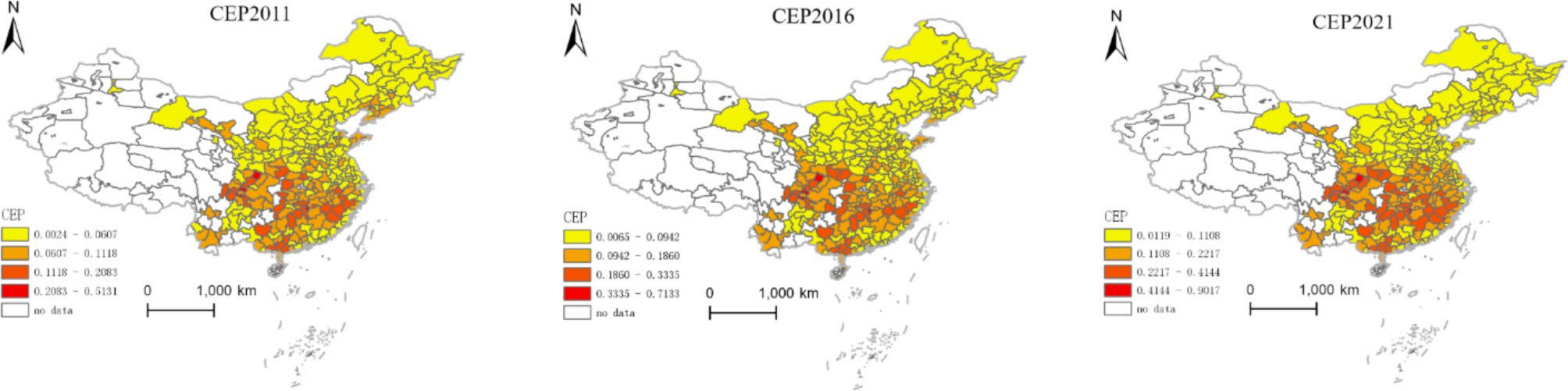
Distribution Map of Urban Carbon Emission Performance. Version: ArcMap 10.8 for desktop URL link: https://www.esri.com/en-us/home.

From Fig. 3, we can observe that in 2011, the overall carbon emission performance generally showed higher levels in the south compared to the north. The regions with high carbon emission performance are concentrated mainly in the southwest and southeast, while the northeast and northwest regions have relatively lower carbon emission performance. In 2021, the overall pattern of higher levels in the south and lower levels in the north persisted, but compared to 2011, the carbon emission performance in the southern regions further improved, with the most significant improvement seen in the Yangtze River Delta region. Furthermore, examining the extreme values of carbon emission performance, in 2011, Ziyang City in Sichuan Province had the highest carbon emission performance (0.51), while Datong City in Shanxi Province had the lowest (0.002), with a difference of approximately 255 times between the two cities. In 2021, Bazhong City in Sichuan Province became the city with the highest carbon emission performance (0.90), while Hulunbeier City in Inner Mongolia Autonomous Region had the lowest (0.11), showing a difference of approximately 8 times. This indicates that over the years, the carbon emission performance in China’s cities has significantly improved, and the spatial disparity in carbon emission performance among cities has gradually decreased.

## Research design

### Model setting

This research utilizes panel data from 284 Chinese cities to investigate the influence of the digital economy on urban carbon emissions, grounded on theoretical analysis and hypotheses. This study used a two-way fixed effects model as the benchmark regression framework for precise analysis, referencing Luan^39^, who applied the same model to examine the impact and processes of the digital economy on inter-city carbon transfer.

1 $$\:{Y}_{it}={\alpha\:}_{0}+{\alpha\:}_{1}{dige}_{it}+{{\alpha\:}_{3}Control}_{it}+{\delta\:}_{i}+{\mu\:}_{t}+{\epsilon\:}_{it}$$

Where, $\:{Y}_{it}$represents the explained variable, indicating the carbon emission intensity and carbon emission performance of city $\:i$ in a given year $\:t$; $\:{dige}_{it}$ is the primary explanatory variable, denoting the level of digital economy development in city $\:i$ in year $\:t$; $\:{Control}_{it}$ are additional control variables influencing urban carbon emissions, encompassing industrial structure (ind), financial development level (fin), social consumption level (cons), economic development level (pgdp), investment level (inv), and urban industrial scale (size). The subscript $\:i$ indicates the city, the subscript $\:t$ signifies the year, $\:{\alpha\:}_{0}$ is the constant term, $\:{\delta\:}_{i}$ is the individual fixed effect, $\:{\mu\:}_{t}$ is the time fixed effect, and $\:{\epsilon\:}_{it}$ is the random error term.

### Clarification of variables

1. Clarified Variables: This study examines two factors. Carbon Intensity (CI) is defined as the ratio of urban carbon emissions to Gross Domestic Product (GDP). A diminished CI value signifies reduced city emissions and acts as an indicator of urban carbon mitigation in this study.This work references the study by Wang et al.^40^ and utilizes data from the Emission Database for Global Atmospheric Research (EDGAR) to illustrate urban carbon emissions, considering data availability, authority, and comprehensiveness. Secondly, Carbon Emission Performance (CEP). At present, researchers employ the Data Envelopment Analysis (DEA) methodology, using energy, capital, and labor as inputs, GDP as the intended result, and CO2 as the undesirable outcome, to evaluate CEP from a neoclassical viewpoint. This strategy prioritizes economic production over non-market societal benefit in the measuring of CEP. This article employs an ecological economics viewpoint, evaluating both economic and social wellbeing, and quantifies CEP using GDP-to-CO2 and URDI-to-CO2 ratios (where URDI denotes Urban Residents’ Development Index). The formulae for measuring are as follows:

2 $$\:CEP=\frac{1}{2}CEE+\frac{1}{2}CSE$$

In this context, CEP denotes urban carbon emission performance, with CEE (economic performance) quantified by the GDP-to-CO2 ratio and CSE (social welfare performance) assessed by the URDI-to-CO2 ratio. This paper develops the URDI based on Wang et al.‘s^41^ framework, utilizing three equally weighted indicators: urban medical capacity (quantified by hospital beds per 10,000 residents), educational attainment (quantified by college students per 10,000 individuals), and income level (quantified by per capita disposable income).

(2) Core Explanatory Variable: Digital Economy Development Level (dige). At now, academics does not possess a standardized metric for the digital economy. The CAICT’s Digital Economy Development Report (2022) defines it through four dimensions: digital industrialization, industrial digitization, data valorization, and digital governance. Considering data availability and following Bai & Zhang^42^ and Zhao et al.^43^, this paper uses the entropy weight method to measure urban digital economy development across four dimensions: digital industrialization, industrial digitization, digital infrastructure, and digital governance (Table 1). Data about industrial robots are obtained from the IFR. This study addresses restrictions in provincial-level data regarding industrial robot installation density and e-commerce sales by employing city-to-province ratios of secondary and tertiary industry added values as weights to create city-level statistics, following the methodology of Fan & Zhao^44^. Government policies with the digital economy illustrate its regulatory function. This article employs the methodology of Xiao et al.^45^ by utilizing Python to analyze government work reports and quantify keywords relevant to the digital economy as a surrogate for urban digital oversight.
(3) This research examines three key mechanism variables. Initially, Green Economic Efficiency (GEE) is assessed utilizing the Super-efficiency slacks-based measure (SBM) model, based on the work of Lin & Tan^25^, with indicators specified in table 2. Secondly, the number of Green Technological Innovations (NGTI) is shown by the natural logarithm of green innovation and utility model patents per 10,000 individuals, as per the study of Feng et al.^46^. Thirdly, the quality of Green Technological Innovations (QGTI) is evaluated using the natural logarithm of green innovation patents per 10,000 individuals, as per Song et al.^47^. Green patent data is derived from the World Intellectual Property Organization’s (WIPO) international patent classification (IPC) codes and is created with patent application and permission data from the National Intellectual Property Administration.
(4) Threshold Variable: Industrial Structure Upgrade (ISU). ISU denotes the transition of the economy from lower-tier to higher-tier industrial structures. This change represents not just an economic shift but also a transition towards more sustainable and efficient manufacturing processes. The selection of ISU as the threshold variable is based on its capacity to represent the transformation of industrial structures, which is expected to significantly impact the efficacy of the digital economy in mitigating carbon emissions and improving efficiency. Consequently, according to Wang et al.^48^, this study computes the ISU coefficient by employing a weighted ratio of primary, secondary, and tertiary industries, where a greater coefficient signifies a more sophisticated industrial structure. By including ISU as a threshold variable, we want to examine how the influence of the digital economy on urban carbon emissions varies throughout different phases of industrial growth. Details of the calculation are provided below:

**Table 1:** Indicator system for digital economy development.

Tier-1 Indicators	Tier-2 Indicators	Tier-3 Indicators	Indicator Meaning	Data Source
Digital Economy	Digital Industrialization	Digital Industry Employees	Employment Proportion in ICS (Information & Computer Services)	China City Statistical Yearbook
		Scale of the Digital Industry	Total Telecom Service Volume	
	Industrial Digitization	Digital Finance Development	Digital Inclusive Finance Index	PKU Digital Financial Inclusion Index
		Application Level of Industrial Robots	Installation Density of Industrial Robots	International Federation of Robotics (IFR)
		Electronic Commerce	E-commerce Sales Volume	E-Commerce Yearbook
	Digital Infrastructure	Broadband Internet Infrastructure	International Internet User Rate per 100 People	China City Statistical Yearbook
		Mobile Internet Infrastructure	Broadband Subscription Rate per 100 People	
	Digital Governance	Digital Regulation	Digital Economy Policy	Compiled Results
		Digital Talent Pool	Number of College-Educated Individuals	China City Statistical Yearbook

**Table 2:** Indicator system for urban green economic efficiency.

First-level Indicators	Second-level Indicators	Indicator Descriptions
Input Indicators	Capital Input	Fixed Asset Capital Stock (10,000 yuan)
	Labor Force	Year-end Employee Count (10,000 persons)
	Water Resource Input	Water Supply Volume (100 million m³)
	Energy Input	Total Societal Electricity Consumption (10,000 kWh)
Desired Output	Economic Output	Regional GDP (billion yuan)
Undesired Output	Environmental Pollution	Industrial Smoke & Dust Emissions (10,000 tons)
		Industrial SO2 Emissions (10,000 tons)
		Industrial Wastewater Discharge (10,000 tons)

3 $$\:ISU=\sum\:_{k=1}^{3}{x}_{k}\times\:k$$

Wherein, $\:{x}_{k}$ represents the fraction of the total output value attributable to the k-th industry, with *k* also serving as the weight factor in the weighted aggregation of the respective industry’s contribution to the total output.

(5) Control Variables: To precisely evaluate the influence of the digital economy on urban carbon emissions and reduce omitted variable bias, we include the following based on prior research: Industrial Structure (ind), Financial Development (fin), Social Consumption (cons), Economic Development (pgdp), Investment Level (inv), and Industrial Scale (size) are quantified by the ratios of tertiary to secondary industry output, financial institution loans to GDP, retail sales to GDP, logarithm of per capita GDP, logarithm of urban fixed asset investment, and logarithm of designated-size industrial enterprises, respectively.

### Data sources and descriptive statistics

The Digital Inclusive Finance Index was developed by the Research Institute at Peking University in collaboration with Ant Financial. Data on industrial robot density was sourced from the IRF, e-commerce sales from the “Electronic Commerce Statistical Yearbook,” and other information from the “China City Statistical Yearbook,” municipal bulletins, and the EPS database. Table 3 displays the descriptive statistics for each variable.

**Table 3:** Descriptive statistics of variables.

Vars.	Interpretation	*N*	M	SD	Med	Min	Max
CI	Carbon emission Intensity	3124	0.4691	0.3749	0.3613	0.0441	2.7201
CEP	Carbon Emission Performance	3124	0.1056	0.0927	0.0798	0.0024	0.9215
dige	Digital Economy	3124	0.0557	0.0554	0.0403	0.0030	0.6423
GEE	Green Economic Efficiency	3124	0.5590	0.2800	0.4675	0.1147	1.6565
NGTI	Number of Green Technology Innovations	3124	0.4265	0.4166	0.2747	0	2.3765
QGTI	Green Tech Innovation Quality	3124	0.1069	0.1689	0.0398	0	1.6385
ISU	Industrial Structure Upgrading	3124	2.3028	0.1459	2.2987	1.8212	2.8357
ind	Industrial Structure	3124	1.0645	0.5982	0.9201	0.1750	5.3482
fin	Financial Development Level	3124	1.0462	0.6119	0.8751	0.1322	7.4502
cons	Social Development Level	3124	0.3850	0.1085	0.3769	3.11E-05	1.0126
pgdp	Economic Development Level	3124	10.7444	0.5701	10.7199	8.7729	13.0557
inv	Investment Level	3124	10.5587	0.7846	10.5885	5.3322	13.0605
size	Industrial Scale	3124	6.6013	1.0834	6.5978	2.9957	9.4748

## Analysis of empirical results

### Benchmark regression analysis

To validate Hypothesis 1, this study uses a panel two-way fixed effects model to examine the digital economy’s impact on urban carbon emissions. Table 4 presents the empirical results. Columns (1) & (2) show significant coefficients (-0.368 & 0.069, *p* < 0.01) without control variables, indicating digital economy development reduces carbon emissions and enhances carbon efficiency. Columns (3) & (4) include control variables: Column (3) shows a significant negative coefficient (-0.228, *p* < 0.01), suggesting a 1% increase in digital economy reduces urban carbon emission intensity by 22.8%. Column (4) shows a significant positive coefficient (0.065, *p* < 0.01), indicating a 1% increase enhances urban carbon efficiency by 6.5%. These findings support Hypotheses 1 & 2, demonstrating the dual benefits of the digital economy in reducing carbon emissions and improving carbon efficiency.

**Table 4:** Benchmark Regression results.

	(1)	(2)	(3)	(4)
	CI	CEP	CI	CEP
dige	-0.368***	0.069***	-0.228***	0.065***
	(-4.500)	(3.006)	(-3.565)	(2.608)
ind			0.045***	0.006***
			(5.847)	(3.131)
fin			0.027**	0.001
			(2.548)	(0.825)
cons			0.040	0.028***
			(1.160)	(4.277)
pgdp			-0.233***	0.068***
			(-12.170)	(13.672)
inv			-0.003	0.002**
			(-0.561)	(2.048)
size			-0.115***	0.014***
			(-8.829)	(6.138)
_cons	0.490***	0.102***	3.691***	-0.753***
	(97.915)	(75.649)	(20.180)	(-16.331)
City FE	YES	YES	YES	YES
Year FE	YES	YES	YES	YES
N	3124	3124	3124	3124
adj. R2	0.938	0.945	0.964	0.962

### Examination of structural effects

Based on the baseline analysis, this study further explores whether the impact of the digital economy on urban carbon emissions differs across various dimensions. Tables 5 and 6 display empirical findings that examine the correlations between several facets of the digital economy and urban carbon emission intensity/performance. Table 5’s Columns (1)-(4) assess the impacts of digital industrialization, industrial digitization, digital infrastructure, and digital governance on carbon intensity. Columns (1), (3), and (4) show significant negative coefficients (*p* < 0.01), signifying that the progression of digital industrialization, governance, and infrastructure may substantially decrease carbon intensity. The potential explanations for this are as follows: Digital industrialization facilitates technological transition, optimizing energy consumption and improving efficiency. Digital governance consolidates and enhances intersectoral data and resources, therefore facilitating carbon reduction. Digital infrastructure has the most substantial influence, presumably owing to its extensive use across economic and social spheres, attaining great integration with the urban economy and society. In contrast, the negative coefficient in Column (2) does not achieve significance, indicating that the influence of industrial digitization on carbon reduction remains insignificant, maybe owing to China’s ongoing industrial structural change and the current phase of digitization growth.

**Table 5:** Dimensional examination results of carbon emission intensity.

	(1)	(2)	(3)	(4)
	CI	CI	CI	CI
dig_ind	-0.770***			
	(-3.398)			
ind_dig		-0.089		
		(-1.145)		
dig_inf			-2.036***	
			(-2.883)	
dig_gov				-0.777***
				(-3.910)
_cons	3.684***	3.681***	3.664***	3.679***
	(19.907)	(19.960)	(19.451)	(19.981)
Controls	YES	YES	YES	YES
City FE	YES	YES	YES	YES
Year FE	YES	YES	YES	YES
N	3124	3124	3124	3124
adj. R2	0.964	0.964	0.964	0.964

**Table 6:** Dimensional examination results of carbon emission performance.

	(1)	(2)	(3)	(4)
	CEP	CEP	CEP	CEP
dig_ind	0.032			
	(0.418)			
ind_dig		0.097***		
		(2.629)		
dig_inf			-0.327*	
			(-1.725)	
dig_gov				0.085*
				(1.676)
_cons	-0.749***	-0.754***	-0.751***	-0.749***
	(-15.764)	(-16.538)	(-16.124)	(-15.756)
Controls	YES	YES	YES	YES
City FE	YES	YES	YES	YES
Year FE	YES	YES	YES	YES
N	3124	3124	3124	3124
adj. R2	0.962	0.962	0.962	0.962

Table 6 Illustrates the effects of digital industrialization, industrial digitization, digital infrastructure, and digital governance on urban carbon emission performance in columns (1) to (4). Regression findings indicate substantial positive coefficients at the 1% and 10% levels for columns (2) and (4), respectively, suggesting that increased urban industrial digitalization and digital governance improve carbon emission performance. Potential factors encompass industrial digitization and improved digital governance, which not only advance new technology research and development, fostering energy-efficient and emission-reducing technologies, enhancing efficiency, and attaining carbon economic performance, but also catalyze the development of new industries, online education, smart city management, and telemedicine, thereby achieving carbon welfare performance. Nonetheless, the coefficient in Column (1), albeit being positive, does not pass significance tests, indicating that the effect of digital industrialization is negligible. This may be due to its dependence on a high-carbon energy framework, which impedes the economic performance of carbon emissions, whilst fostering economic development. Furthermore, the environmental costs of digital industrialization, such as electronic trash, may impose immediate strain on the environment, affecting carbon welfare performance. Conversely, the markedly negative value in Column (3) signifies the detrimental impact of digital infrastructure. Potential explanations encompass: the building of digital infrastructure utilizes resources, leading to carbon emissions from both manufacture and transportation. Moreover, abbreviated maintenance cycles diminish resource efficiency and elevate the carbon impact. Although it improves urban service quality and accessibility, hence enhancing carbon performance, economic and knowledge constraints intensify the digital gap and inequality. This hinders some towns and populations from accessing digital advantages such as education, healthcare, and employment prospects, hence adversely affecting carbon welfare performance.

### Robustness checks

To ensure the reliability of the research findings, this paper employs the following methods to conduct robustness checks:

1. Variable Substitution.

Step 1: We replace the core explanatory variable, considering that measurement differences may bias the results. Following Pan et al.^49^, we use PCA to remeasure urban digital economic development. Table 7, Columns (1) and (2), show the PCA-based digital economy regression results. Column (1)’s significantly negative coefficient (at the 1% level) aligns with the benchmark results, indicating that the digital economy reduces urban carbon emission intensity. Column (2)’s significantly positive coefficient (at the 5% level) also matches the benchmarks, suggesting that the digital economy enhances urban carbon emission performance.

Step 2: We substitute the explained variable. The benchmark regression uses carbon emission intensity. For verification, we adopt Dong et al.‘s^50^ per capita carbon emissions. Using Wu and Guo’s^51^ method, we remeasure urban carbon emissions and performance. Table 7, Columns (3) and (4), display the results. Column (3)’s significantly negative coefficient (at the 1% level) confirms that the digital economy lowers urban per capita carbon emissions. Column (4)’s significantly positive coefficient (at the 1% level) shows that the digital economy boosts urban carbon emission performance.

Step 3: For robustness, we substitute both variables. PCA measures digital economic development, and per capita carbon emissions replace intensity. Carbon emission performance is recalculated accordingly. Table 7, Columns (5) and (6), present the results. Column (5)’s significantly neg.

**Table 7:** Robustness check I.

	Step 1		Step 2		Step 3	
	(1)	(2)	(3)	(4)	(5)	(6)
	CI	CEP	PCO2	CEP	PCO2	CEP
dige	-0.032***	0.004**	-2.913***	0.307***	-0.069***	0.007**
	(-4.400)	(2.438)	(-8.529)	(5.304)	(-3.257)	(2.234)
_cons	4.007***	-0.788***	5.064***	-0.158***	5.589***	-0.210***
	(18.957)	(-15.878)	(9.856)	(-3.026)	(10.098)	(-3.846)
Controls	YES	YES	YES	YES	YES	YES
City FE	YES	YES	YES	YES	YES	YES
Year FE	YES	YES	YES	YES	YES	YES
N	3124	3124	3124	3124	3124	3124
adj. R2	0.965	0.962	0.988	0.920	0.988	0.917

(2) Exclusion of Municipalities Directly Under the Central Government: Due to significant differences in economic, financial development, and carbon emissions between municipalities directly under the central government (Beijing, Tianjin, Shanghai, Chongqing) and others, this paper excludes their samples and re-runs regression tests. The results in Table 8, Columns (1) and (2), show that excluding these municipalities, the digital economy coefficients are significantly negative and positive at the 1% level, aligning with benchmark results, confirming robustness.
(3) Winsorization of Control Variables: This paper applies a 1% winsorization to mitigate extreme value impacts on estimation. Table 8, Columns (3) and (4), show that post-winsorization, digital economy coefficients are significantly negative and positive at the 1% level, confirming its urban carbon reduction and efficiency enhancement effects.
(4) Sample Period Adjustment: Given China’s rapid digital economy growth since 2015, this paper adjusts the sample period to 2015–2021 and re-estimates. Table 9, Columns (1) and (2), show that post-adjustment, digital economy coefficients are significantly negative and positive at the 1% level, aligning with benchmark results.
(5) Elimination of Low-Carbon Pilot Interference: To accurately assess digital economy’s impact on urban carbon emissions, this paper excludes low-carbon pilot cities and re-tests. Table 9, Columns (3) and (4), show that post-exclusion, digital economy coefficients remain significantly negative and positive at the 1% level, reinforcing paper conclusions.
(6) Endogeneity Test.

- Two-stage least squares (2SLS).

**Table 8:** Robustness check II.

	Exclusion of Municipalities Directly Under the Central Government		Winsorization of Control Variables	
	(1)	(2)	(3)	(4)
	CI	CEP	CI	CEP
dige	-0.352***	0.124***	-0.200***	0.061***
	(-3.600)	(3.724)	(-3.357)	(2.633)
_cons	3.714***	-0.758***	3.634***	-0.794***
	(20.195)	(-16.789)	(20.891)	(-22.360)
Controls	YES	YES	YES	YES
City FE	YES	YES	YES	YES
Year FE	YES	YES	YES	YES
N	3080	3080	3124	3124
adj. R2	0.964	0.963	0.964	0.963

**Table 9:** Robustness check III.

	Adjustment of Time Window		Exclusion of Low-carbon Cities	
	(1)	(2)	(3)	(4)
	CI	CEP	CI	CEP
dige	-0.286***	0.082***	-0.236***	0.059**
	(-3.714)	(3.282)	(-3.575)	(2.297)
_cons	4.223***	-0.777***	3.712***	-0.754***
	(16.826)	(-19.708)	(20.087)	(-16.141)
Controls	YES	YES	YES	YES
City FE	YES	YES	YES	YES
Year FE	YES	YES	YES	YES
N	1988	1988	3069	3069
adj. R2	0.980	0.988	0.964	0.963

This study employs first- and second-order lags of the digital economy as instrumental variables to mitigate endogeneity in regression outcomes, in accordance with Guo et al.^52^. The delays are associated with present digital economy levels owing to historical progression but affect current carbon emissions independently, fulfilling exogeneity criteria. Table 10 displays the results of the endogeneity test utilizing two-stage least squares (2SLS). Columns (1) and (4) exhibit substantial positive coefficients for these delays at the 1% significance level. Initial-stage assessments dismiss underidentification (Kleibergen-Paap rk LM = 11.25, *p* = 0.000) and issues of poor instruments (Cragg-Donald Wald F = 9246.87 > 16.38). Second-stage tests dismiss underidentification (Kleibergen-Paap rk LM = 10.17, *p* = 0.000) and validate the robustness of the instruments (Cragg-Donald Wald F = 4299.46 > 16.38). Columns (2)-(3) and (5)-(6) display the results of the second-stage regression, revealing that the coefficients for the digital economy are significantly negative and positive at the 1% level, respectively. This indicates that, after resolving endogeneity concerns, the digital economy continues to exert a carbon emission reduction effect and improves urban efficiency.

**Table 10:** Endogeneity Test results(2SLS).

	(1)	(2)	(3)	(4)	(5)	(6)
	dige	CI	CEP	dige	CI	CEP
L.dige	0.930***					
	(91.21)					
L2.dige				0.889***		
				(61.81)		
dige		-0.399***	0.091***		-0.506***	0.130***
		(-4.49)	(4.09)		(-4.88)	(5.13)
Controls	YES	YES	YES	YES	YES	YES
City FE	YES	YES	YES	YES	YES	YES
Year FE	YES	YES	YES	YES	YES	YES
Cragg-Donald Wald F	9246.87 [16.38]			4299.46 [16.38]		
Kleibergen-Paap Wald rk F statistic	11.25 [0.000]			10.17 [0.000]		
N	2840	2840	2840	2556	2556	2556

b. Instrumental Variables(IV).

This article employs external instrumental variables to mitigate the influence of endogenous issues on study outcomes. According to Zhao Tao et al.^43,53^, the cross-product of the digital economy from the preceding year and the historical data of post and telecommunications from 1984 was employed as the instrumental variable (IV). Table 11 illustrates the application of a two-stage regression approach to mitigate endogenous issues. The findings from the initial phase demonstrate that the estimated coefficient of the instrumental variable and dige is considerably positive, and the chosen instrument is justifiable. The findings from the second stage indicated that the regression coefficient of dige on CI was − 0.354, which was significantly negative at the 1% level. Conversely, the regression coefficient of dige on CEP was 0.196, significantly positive at the 1% level. This suggests that the conclusions of this article remain robust after addressing endogenous concerns and successfully passing the causality test. The p-value of the LM test is 0.000, indicating significance at the 1% level, while the Wald·F statistic is 857.860, above the crucial value of 16.38 at the 10% level of the Stock-Yogo weak identification test. The null hypotheses of inadequate identification and weak identification of instrumental variables are rejected, signifying that instrumental variables.

**Table 11:** Endogeneity Test results (IV).

	(1)	(2)	(3)
	The first stage	The second stage	The second phase
	dige	CI	CEP
IV	0.001^***^		
	(29.289)		
dige		-0.354^***^	0.196^***^
		(-3.100)	(6.756)
ind	0.006^***^	0.050^***^	0.007^***^
	(3.142)	(8.101)	(4.348)
fin	-0.002	0.031^***^	0.001
	(-1.500)	(5.473)	(0.513)
cons	-0.003	0.027	0.031^***^
	(-0.869)	(1.220)	(5.473)
pgdp	0.006^**^	-0.230^***^	0.064^***^
	(2.504)	(-22.047)	(24.344)
inv	-0.003^***^	-0.003	0.002^*^
	(-4.121)	(-0.922)	(1.891)
size	0.003^**^	-0.112^***^	0.014^***^
	(2.515)	(-14.993)	(7.632)
Year	Yes	Yes	Yes
City	Yes	Yes	Yes
*N*	2840	2840	2840
R^2^	0.957	0.573	0.672
Kleibergen-Paap rk LM statistic		72.148	
		(0.000)	
Kleibergen-Paap rk Wald F statistic		857.860	
		(16.38)	

### Heterogeneity analysis

1. Regional Heterogeneity Analysis.

Given China’s vast territory and significant geographical disparities among cities, leading to varied economic development stages and digital economy progress, along with differing carbon emissions, this study classifies 284 sample cities into East, Central, and West using the National Bureau of Statistics’ regional standard. The aim is to explore regional variations in the impact of digital economy on urban carbon emissions. Regression results are summarized in Table 12. In the Central region (Column 3), the digital economy coefficient (-0.789) is significantly negative at the 1% level, indicating a substantial carbon reduction effect. This could be due to the region’s reliance on traditional industries, where digitalization facilitates transformation and upgrade, enhancing energy efficiency and reducing emissions. In contrast, the Eastern (Column 1) and Western (Column 5) regions show non-significant coefficients (-0.040 and − 0.047, respectively). Eastern cities, with advanced economies and infrastructure, may have already implemented effective carbon reduction strategies, reducing the marginal benefit of digital economy. In the West, despite potential for digital-aided carbon reduction, economic, infrastructural, and technological gaps hinder significant impacts. Interestingly, the Eastern region (Column 2) exhibits a significant positive coefficient (0.092 at 1% level) for digital economy’s enhancement of carbon emission performance. This could be explained by the region’s robust economy aligning with digital development, fostering efficient models that boost output while reducing emissions per unit, and improving resident welfare through job creation and social services. Columns 4 and 6 reveal non-significant coefficients (0.055 and 0.072) for the Central and Western regions, respectively. While digital economy progress is evident, it lags behind the East, failing to significantly reduce emissions from economic activities. Additionally, less developed digital infrastructure in these regions hinders the realization of carbon emission welfare effects.

**Table 12:** Regional Heterogeneity Test.

	Eastern Region		Central Region		Western Region	
	(1)	(2)	(3)	(4)	(5)	(6)
	CI	CEP	CI	CEP	CI	CEP
dige	-0.040	0.092***	-0.789***	0.055	-0.047	0.072
	(-0.796)	(3.201)	(-3.869)	(0.955)	(-0.241)	(1.363)
_cons	2.857***	-0.393***	4.292***	-0.834***	3.375***	-0.972***
	(13.202)	(-5.482)	(12.683)	(-18.355)	(12.113)	(-11.219)
Controls	YES	YES	YES	YES	YES	YES
City FE	YES	YES	YES	YES	YES	YES
Year FE	YES	YES	YES	YES	YES	YES
N	1100	1100	1100	1100	924	924
adj. R2	0.971	0.950	0.968	0.966	0.962	0.964

(2) Heterogeneity Analysis According to Urban Hierarchy.

Central cities in China are crucial for regional economic growth and significantly affect the relationship between digital finance and urban carbon emissions across various city tiers. This research classifies cities into central cities (comprising municipalities directly governed by the federal government, provincial capitals, and deputy provincial capitals) and non-central cities to examine this heterogeneity. The regression outcomes (displayed in Table 13) indicate the following: In urban centers (Column 1), the coefficient of the digital economy (0.250, significant at the 1% level) suggests that it elevates carbon emissions, likely attributable to heightened economic development and population density, which intensify energy demand from digital infrastructure. In contrast, in non-central cities (Column 3), the coefficient (-0.520, significant at the 1% level) indicates that the digital economy decreases carbon emissions, perhaps due to their reduced economic size and lower population density, which lead to decreased energy consumption and enhanced energy efficiency. Moreover, in urban centers (Column 2), the digital economy improves carbon emission performance (coefficient 0.086, significant at the 5% level), likely due to efficiency advancements from digital technology and appealing digital lifestyles for highly trained inhabitants. In non-central cities (Column 4), the digital economy negatively impacts carbon emission performance (coefficient − 0.127, significant at the 1% level), likely due to less compatibility with the digital economy, limited technological penetration, and superficial application depth.

**Table 13:** Heterogeneity test based on Urban Hierarchy.

	Central Cities		Non-Central Cities	
	(1)	(2)	(3)	(4)
	CI	CEP	CI	CEP
dige	0.250***	0.086**	-0.520***	-0.127***
	(3.079)	(2.469)	(-3.398)	(-2.813)
_cons	1.908***	-0.624***	3.903***	-0.786***
	(6.088)	(-3.805)	(19.509)	(-17.197)
Controls	YES	YES	YES	YES
City FE	YES	YES	YES	YES
Year FE	YES	YES	YES	YES
N	385	385	2739	2739
adj. R2	0.957	0.948	0.965	0.964

(3) Analysis of Heterogeneity According to Urban Categories.

Historically, resource-dependent cities in China have been substantial carbon emitters. This research, derived from the “National Plan for the Sustainable Development of Resource-based Cities (2013–2020)” by the State Council, classifies cities as resource-based or non-resource-based to investigate the varied effects of the digital economy on urban carbon emissions. The regression findings shown in Table 14 indicate:

In resource-based cities (Columns 1 & 3), the digital economy exhibits a markedly negative coefficient (-1.435 and − 0.221, both at the 1% significance level), indicating a more substantial carbon reduction effect in resource-based cities due to the larger absolute value. This results from the digital economy’s capacity for industrial optimization and enhancements in energy efficiency within high-carbon sectors. Furthermore, it augments carbon emission monitoring, hence enhancing the efficacy of carbon reduction efforts.

In non-resource-based cities (Columns 2 & 4), the digital economy has a markedly negative coefficient (-0.251, at the 1% significance level) in Column 2, whereas in Column 4, it presents a considerably positive coefficient (0.046, at the 10% significance level). This suggests that the digital economy enhances carbon emission performance in non-resource-based cities while adversely affecting resource-based cities. This may arise from the greater compatibility of the digital economy with the developmental paradigm of non-resource-based cities.

In resource-dependent cities, the digital transformation of high-carbon industry encounters technological and economic obstacles. Although digital technology enhances industrial efficiency, these sectors remain significantly dependent on energy, and improvements may produce increased carbon emissions, leading to adverse carbon emission outcomes at this juncture. Moreover, although the digital economy may ultimately improve carbon emission metrics in resource-dependent cities over time, immediate industrial optimization might result in employment insecurity and heightened societal costs, adversely affecting welfare outcomes associated with carbon emissions.

**Table 14:** Heterogeneity test based on city types.

	Resource-Based Cities		Non-Resource-Based Cities	
	(1)	(2)	(3)	(4)
	CI	CEP	CI	CEP
dige	-1.435***	-0.251***	-0.221***	0.046*
	(-3.299)	(-2.723)	(-3.593)	(1.869)
_cons	4.935***	-0.787***	2.907***	-0.713***
	(19.893)	(-21.002)	(13.382)	(-10.390)
Controls	YES	YES	YES	YES
City FE	YES	YES	YES	YES
Year FE	YES	YES	YES	YES
N	1078	1078	2046	2046
adj. R2	0.970	0.965	0.950	0.962

(4) Heterogeneity Analysis Stratified by Digital Economy Structure.

Based on the quartile (Q1 at 1/4 and Q3 at 3/4) division of the Digital Economy (Dige) index, cities are categorized into low, medium, and high levels to investigate whether there are differences in carbon emission reduction (CI) and carbon emission performance (CEP) across these levels. As illustrated in Table 15, the heterogeneous regression results for the digital economy reveal that, in terms of its impact on carbon emission intensity, under low digital economy conditions (Column 1), carbon emissions increase; under medium digital economy conditions (Column 2), carbon emissions decrease; and under high digital economy conditions (Column 3), carbon emissions decrease even more significantly. This indicates that the intensity of the digital economy does reduce carbon emission intensity and exhibits notable heterogeneity. In terms of its impact on carbon emission performance, under low digital economy conditions (Column 4), the effect is insignificant; under medium digital economy conditions (Column 5), the effect is also insignificant; however, under high digital economy conditions (Column 6), carbon emission performance improves. This suggests that the digital economy does enhance carbon emission performance and demonstrates clear heterogeneity. Therefore, different levels of digital economy exhibit distinct heterogeneities and have different effects.

**Table 15:** Heterogeneity test based on digital economy structure.

	(1)	(2)	(3)	(4)	(5)	(6)
	low	medium	high	low	medium	high
	CI	CI	CI	CEP	CEP	CEP
dige	0.111^**^	-2.482^*^	-0.845^**^	0.041	-0.415	0.155^**^
	(2.561)	(-1.956)	(-2.324)	(0.876)	(-1.246)	(2.382)
ind	-0.000	0.037	0.050^***^	0.010^*^	-0.015^***^	0.010^***^
	(-0.044)	(1.128)	(4.712)	(1.762)	(-2.638)	(5.215)
fin	0.042^***^	0.010	0.036^*^	-0.007^*^	-0.000	0.002
	(7.004)	(0.742)	(1.681)	(-1.942)	(-0.072)	(1.460)
cons	-0.002	0.170^**^	-0.086^*^	0.045^*^	0.022	0.042^***^
	(-0.103)	(2.164)	(-1.787)	(1.675)	(0.891)	(8.424)
pgdp	-0.087^***^	-0.363^***^	-0.362^***^	0.027^***^	0.047^***^	0.077^***^
	(-8.293)	(-6.649)	(-13.309)	(3.942)	(4.712)	(17.324)
inv	-0.010^***^	-0.023	0.024^***^	0.003	0.009^**^	0.001
	(-2.955)	(-1.379)	(2.680)	(1.107)	(2.192)	(0.935)
size	-0.076^***^	-0.103^***^	-0.091^***^	-0.002	0.010	0.004^*^
	(-8.779)	(-4.492)	(-3.879)	(-0.794)	(1.017)	(1.742)
_cons	1.896^***^	5.129^***^	4.672^***^	-0.250^***^	-0.530^***^	-0.777^***^
	(15.352)	(8.880)	(17.744)	(-3.071)	(-3.483)	(-18.107)
*N*	732	753	1558	781	753	1558
r2	0.983	0.988	0.980	0.146	0.981	0.987
Year	Yes	Yes	Yes	Yes	Yes	Yes
City	Yes	Yes	Yes	Yes	Yes	Yes

## Examination of mechanisms

This study explores the impact of digital economy development on urban carbon emissions, focusing on green economic efficiency, green technological innovation quantity, and quality. Following Jiang ‘s^54^ approach to address potential endogeneity issues, the study adopts the following framework:

Firstly, mechanism variables (M) are selected for green development and green innovation effects: Green economic efficiency for the former, and green technological innovation quantity and quality for the latter.

Secondly, the study demonstrates the influence of these mechanism variables on urban carbon emissions (M→Y), based on existing literature.

Lastly, the impact of the core explanatory variable (digital economy development) on these mechanism variables (X→M) is examined using a benchmark regression model.

4 $$\:{M}_{it}={\alpha\:}_{0}+{\alpha\:}_{1}{dige}_{it}+{{\alpha\:}_{3}\sum\:Control}_{it}+{\delta\:}_{i}+{\mu\:}_{t}+{\epsilon\:}_{it}$$

Among them, $\:{M}_{it}$represents the mechanism variable. If the coefficient of $\:{\alpha\:}_{1}$ is significant, it indicates that the development of the digital economy has an impact on the mechanism variable.

1. Channel for Green Development Effects.

This study examines the impact of the digital economy on urban carbon emissions through green efficiency in the context of green development.Table 16 shows the regression results. Column (1) reveals a significant positive coefficient of 0.487 for the digital economy at the 5% significance level, indicating a 48.7% enhancement in urban green economic efficiency for each 1% rise in the digital economy. This fosters urban green growth, facilitating carbon reduction and efficiency, hence validating Hypotheses H1 and H2.

(2) Channel of Green Innovation Effects.

This study examines the impact of the digital economy on urban carbon emissions via the lens of green innovation, taking into account both the number and quality of green technological advancements.Table 16 presents the regression results. Column (2) displays a notable positive coefficient of 2.709 for the digital economy at the 1% significance level, signifying a 2.709% rise in urban green technology innovation quantity for each 1% increase in the digital economy. Column (3) indicates a coefficient of 1.371, significant at the 1% level, implying a 1.371% enhancement in quality for each 1% increase. This validates Hypothesis H3.

**Table 16:** Mechanism validation.

	(1)	(2)	(3)
	GEE	NGTI	QGTI
dige	0.481**	2.709***	1.371***
	(2.124)	(13.117)	(10.722)
_cons	-1.183***	0.911***	0.163**
	(-4.753)	(5.498)	(2.399)
Controls	YES	YES	YES
City FE	YES	YES	YES
Year FE	YES	YES	YES
N	3124	3124	3124
adj. R2	0.735	0.928	0.909

## Additional analysis

Analysis of Threshold Effects: This study used a panel threshold model to examine the effect of the digital economy on urban carbon emissions, focusing on the possible threshold effect related to industrial structure upgrading and its nonlinear effects. According to Hansen’s^55^ study, the panel threshold model is formulated as follows:

5 $$\:Y_{{it}} = \eta \:_{0} + \eta \:_{1} dige*I\left( {Z \ll \:\lambda \:_{1} } \right) + \eta \:_{2} dige*I\left( {Z > \lambda \:_{1} } \right) + \gamma \:_{6} Control_{{it}} + \delta \:_{i} \mu \:_{t} + \varepsilon \:_{{it}}$$

Z represents the threshold variable, I denotes the indicator function, and λ signifies the threshold value. The Bootstrap technique replicates the asymptotic distribution of the F statistic, producing P-values and confidence intervals. Threshold effects are computed for F-values and P-values based on 3,200 repetitions (Table 17). The findings indicate that industrial structure upgrading successfully meets the single-threshold test at both 5% and 10% significance levels, with thresholds of 2.1033 and 2.1706, hence confirming Hypothesis H3. A single-threshold model is used to examine the threshold influence of the digital economy on urban carbon emissions. CI denotes Carbon Intensity, whereas CP signifies Carbon Performance.

**Table 17:** Threshold estimation and test results.

Vars.	Threshod Types	Threshod Value	Statistical Quantity		95% Confidence Interval	Critical Value(%)		
			F-value	P-value		10	5	1
CI	Single	2.1033	43.18	0.0384	[2.1018–2.1058]	32.4058	40.3353	57.0512
	Double	2.386	9.38	0.7606	—			
CP	Single	2.1706	40.05	0.0756	[2.1640–2.1718]	36.9879	46.2765	73.7957
	Double	2.4684	11.91	0.6744	—			

**Fig. 4:**
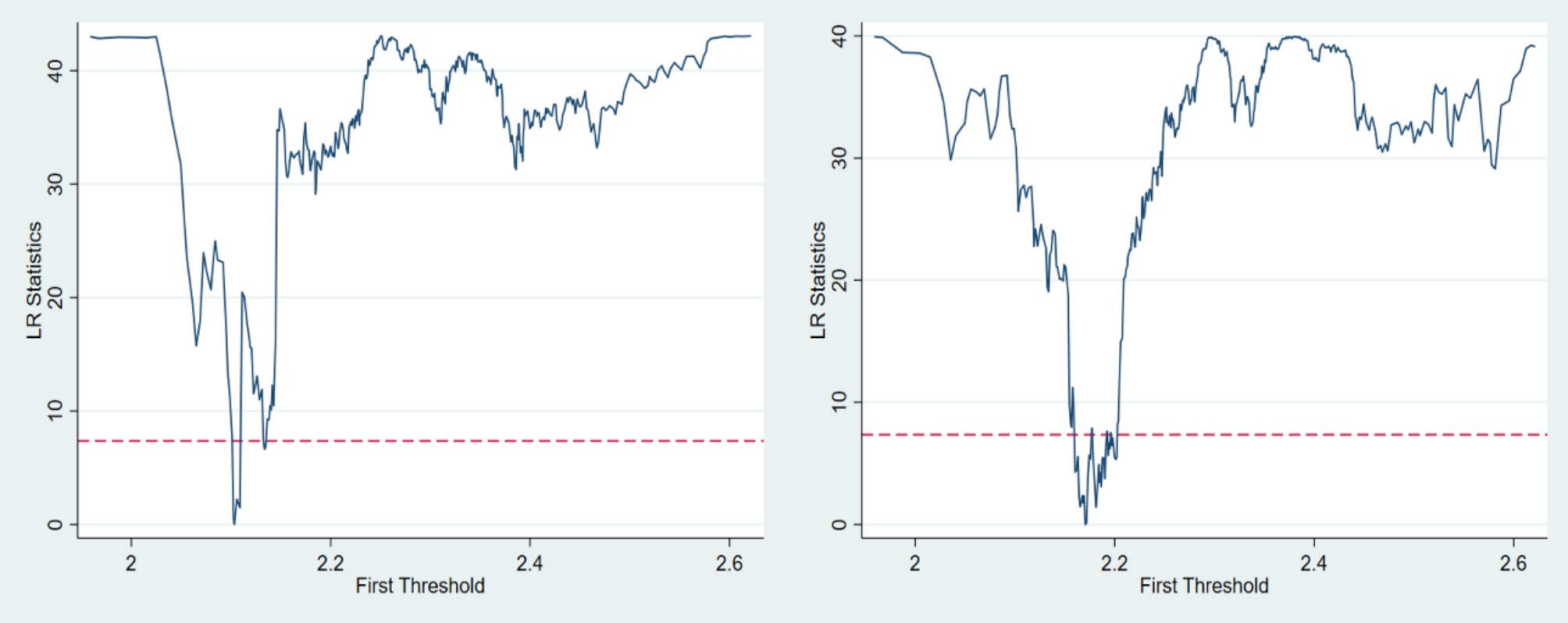
Likelihood function plot.

Figure 4 shows the likelihood function plot of model threshold estimates for carbon emission intensity and performance, with 95% CI distributions. Thresholds of 2.1033 and 2.1706 are at minima. The dashed line is the 1% significance level LR critical value. LR statistics for thresholds are below this value, with narrow 95% CIs, indicating genuine threshold effects and significant identification. Based on this, Table 18 presents the threshold regression results.

**Table 18:** Threshold regression results.

	(1)	(2)
	CI	CEP
dige#isu[isu ≤ 2.1033]	-2.240***	
	(-7.190)	
dige#isu[isu>2.1033]	-0.276***	
	(-3.986)	
dige#isu[isu ≤ 2.1706]		-0.190***
		(-3.744)
dige#isu[isu>2.1706]		0.105***
		(6.054)
_cons	4.103***	-0.793***
	(55.229)	(-41.975)
Controls	YES	YES
City FE	YES	YES
Year FE	YES	YES
N	3124	3124
adj. R2	0.531	0.639

Based on Table 17, the digital economy’s impact on urban carbon emissions is nonlinear, contingent on industrial structure upgrading. Regression results in column (1) show significant negative coefficients for the digital economy at the 1% level under a single threshold of industrial structure upgrading. This indicates a nonlinear transformation in the digital economy’s carbon emission reduction effect, weakening as industrial structure upgrading progresses. Specifically, beyond a threshold of 2.1033, the digital economy’s carbon reduction effect diminishes, though still significant. This may be due to a shift from high- to low-carbon industries, reducing the digital economy’s contribution to urban carbon reduction. According to column (2) regression results, under a single threshold of industrial structure upgrading, the digital economy exhibits a significant U-shaped relationship with urban carbon emission performance at the 1% level. Specifically, a low level of industrial structure upgrading negatively impacts urban carbon emission performance via the digital economy, but surpassing a threshold of 2.1706 reverses this to a positive influence. Initially, as industrial structure upgrading rises below 2.1706, the digital economy hinders urban carbon emission performance due to mismatch. However, as upgrading improves, compatibility increases, enabling the digital economy to enhance resource allocation efficiency and steadily improve carbon emission performance. This is because advanced productive forces like the digital economy require compatible advanced industries.

## Geographically and temporally weighted regression (GTWR)

To further affirm the impact of the digital economy on carbon emissions, we coupled digital economy intensity (CI) and digital economy productivity (CEP) into a novel variable termed the carbon emission coupling coordination degree (syeff). In the geographically and temporally weighted regression (GTWR) analysis, syeff was utilized as the new dependent variable, while the previously defined digital economy (Dige) served as the explanatory variable. The results, presented in Table 19, indicate significance at the 95% confidence level.

**Table 19:** GTWR of digital economy on carbon emission coupling.

Variable	Est.	SE	T(Est/SE)	*P*-value
Intercept	0.000	0.017	0.000	1.000
dige	0.055	0.028	1.998	0.046
ind	0.133	0.023	5.857	0.000
fin	-0.140	0.022	-6.304	0.000
cons	0.086	0.019	4.462	0.000
pgdp	-0.091	0.027	-3.355	0.001
size	0.193	0.024	8.056	0.000
inv	0.233	0.021	10.968	0.000

In addition, we also produced spatiotemporal dynamic differentiation maps to illustrate the results. The findings are presented as follows:

**Fig. 5:**
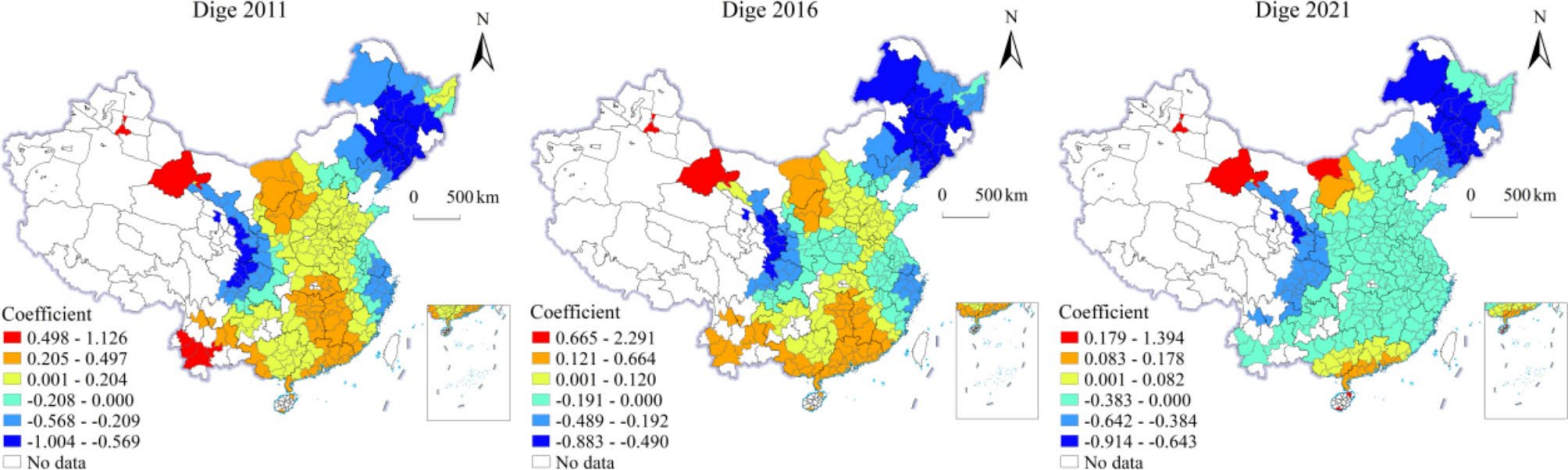
Temporal-spatial impact of digital economy on coupling coordination. Version: ArcMap 10.8 for desktop URL link: https://www.esri.com/en-us/home.

As illustrated in Fig. 5, the regression coefficients of the Digital Economy (Dige) on the coupling coordination degree of carbon emissions (syeff) in 2011 exhibited a spatial pattern of higher values in the west and south compared to lower values in the east and north. Positive impacts were concentrated in central inland cities and southern coastal cities, with the highest positive coefficients primarily observed in northwestern cities of Gansu Province and southwestern cities of Yunnan Province. Conversely, negative effects were centered in some central cities, eastern coastal cities, and northeastern cities, with the most significant negative coefficients found in parts of Sichuan and Gansu Provinces, as well as in selected cities of Northeast China. When comparing 2016 with 2011, the positive effects weakened in some central and Yunnan cities, while the negative effects intensified in northeastern cities. By 2021, relative to 2016, most central and coastal cities shifted towards negative effects, whereas the negative effects diminished in northeastern cities and positive effects strengthened in some regions of Inner Mongolia. In 2011, Jiuquan City exhibited the highest positive effect (1.112), and the city with the highest negative effect had a coefficient of -1.004; whereas in 2021, Urumqi demonstrated the highest positive effect (1.394), and the city with the highest negative effect recorded a coefficient of -0.914. These observations indicate that, over the years, there has been notable regional heterogeneity in the impact of the digital economy on carbon emissions among Chinese cities, with the disparity in these effects gradually narrowing.

## Conclusions and recommendations

### Research conclusions

Using Chinese city-level panel data from 2011 to 2021, this paper empirically examines the impact of digital economy development on urban carbon emissions and concludes as follows:

1. The digital economy has dual effects on urban carbon emissions: reducing emissions and enhancing efficiency. A 1% increase in its development level can lower urban carbon intensity by 22.8% and improve carbon emission performance by 6.5%. These results are confirmed by robustness and endogeneity tests. Structurally, digital industrialization, infrastructure, and governance reduce carbon intensity, while industrial digitization and governance enhance carbon performance. However, digital infrastructure negatively impacts carbon efficiency, and digital industrialization’s effect is insignificant.
2. The impact of the Digital Economy (Dige) on carbon emission coordination (syeff) exhibits significant spatial heterogeneity. Between 2011 and 2021, the positive effects observed in western and southern China gradually diminished and shifted towards northeastern regions and Inner Mongolia, while negative effects became increasingly prominent in central and eastern coastal cities. Furthermore, across cities with varying levels of digital economy development, the influence of the digital economy on carbon emission intensity and performance also demonstrates notable heterogeneity: At low levels of digital economy, carbon emission intensity increased with no significant change in performance; at medium levels, carbon emission intensity decreased, yet performance remained insignificant; and at high levels of digital economy, both carbon emission intensity and performance saw marked improvements, with inter-regional differences gradually narrowing over time.
3. The digital economy significantly and nonlinearly impacts urban carbon emissions. It enhances urban green economic efficiency and green technological innovation, fostering green development and innovation effects, which promote emission reduction and efficiency. Additionally, urban industrial structure upgrading is a threshold factor influencing the digital economy’s effect on carbon emissions. As industrial structure upgrading improves, the digital economy’s effect on carbon reduction weakens, while its effect on carbon efficiency shifts from inhibitory to promotional.
4. The impact of the digital economy on urban carbon emissions varies across locations, city tiers, and types: ① Geographically, it significantly reduces emissions in central cities but enhances efficiency in eastern cities only. ② By city tier, it increases both emissions and efficiency in central cities, but decreases both in non-central cities. ③ Regarding urban types, the digital economy reduces both emissions and efficiency in resource-based cities, but cuts emissions while boosting efficiency in non-resource-based cities.

### Mitigation strategies and recommendations

1. The digital economy markedly decreases urban carbon emissions and improves carbon efficiency, as evidenced by theoretical and empirical studies.Therefore, enhancing its development to realize its potential is essential. Structural effects assessments reveal significant influences on urban carbon emissions. Therefore, it is essential to prioritize digital industrialization, infrastructure, and governance for urban carbon reduction, while assuring synchronized industrial digitalization. Furthermore, utilizing industrial digitization and governance to enhance carbon efficiency, while promoting their synergistic advancement alongside digital industrialization and infrastructure, will maximize their potential and convert adverse effects into beneficial outcomes.
2. Utilizing green development and innovation, the digital economy can diminish urban carbon emissions and improve efficiency. Emphasizing its function in enhancing urban green economic efficiency can harness its potential to optimize production, energy consumption, and sustainable consumption, resulting in holistic advancements and supporting green development. Simultaneously, leveraging its advocacy for green technological innovation, we must get support in technology, financing, talent development, and market growth to advance innovation and promote a green impact, facilitating carbon reduction and efficiency improvement.
3. The emphasis should be on the threshold effect of industrial structure enhancement in mediating the influence of the digital economy on urban carbon emissions. Research indicates that when advancements in technology escalate, the digital economy transitions from obstructing to enhancing urban carbon efficiency. Although its effect on decrease diminishes, it remains superior to the baseline. Consequently, the robust advancement of high-tech, emergent, advanced manufacturing, and future sectors is essential for optimizing structural integrity to augment the digital economy’s impact on urban carbon reduction and efficiency.
4. Consideration must be given to the varied effects of the digital economy on urban carbon emissions. Initially, targeted strategies must address the strengths and weaknesses of cities: Improve carbon efficiency in the developed eastern regions and prioritize emission reduction in the less developed middle and western regions. Secondly, cities of varying tiers should focus distinct impacts of the digital economy: core cities ought to investigate digital avenues for carbon reduction, whereas non-central towns should contemplate innovative strategies for enhancing carbon efficiency. Ultimately, both resource-based and non-resource-based cities, the digital economy substantially facilitates emission reduction, however it hampers efficiency in resource-based cities. Consequently, non-resource-based cities can stimulate advancements in the digital economy of resource-based cities, thereby improving their carbon efficiency.

## Data Availability

The datasets used and analyzed during the current study available from the corresponding author on reasonable request.
